# Rupture sous-cutanée traumatique du ligament quadricipital chez un sujet jeune

**DOI:** 10.11604/pamj.2015.21.40.6698

**Published:** 2015-05-21

**Authors:** Kevin Parfait Bienvenu Bouhelo-Pam, Mohamed Shimi, Mohamed El Idrissi, Abdelhalim El Ibrahimi, Abdelmajid El Mrini

**Affiliations:** 1 Service de Chirurgie Ostéo-articulaire B4, Fès, Maroc

**Keywords:** Rupture spontanée, tendon quadricipital, traumatisme, genou, spontaneous rupture, quadriceps tendon, trauma, knee

## Abstract

La rupture sous-cutanée traumatique du tendon quadricipital est exceptionnelle. Nous présentons un patient de 19 ans victime d'un accident de sport occasionnant cette blessure. La réparation chirurgicale puis la rééducation fonctionnelle ont permis une récupération complète des fonctions du genou et de la marche.

## Introduction

La rupture sous-cutanée du tendon quadricipital est rare. Elle s'observe souvent chez des patients ayant passé la quarantaine dans un contexte de maladie systémique préexistant [1]. Elle a aussi été rapportée après prise chronique de stéroïdes ainsi que chez les patients obèses [2]. Notre étude rapporte une observation clinique concernant une rupture traumatique chez un sujet jeune avec pour but de contribuer à en améliorer la prise en charge.

## Patient et observation

**Figure 1:**
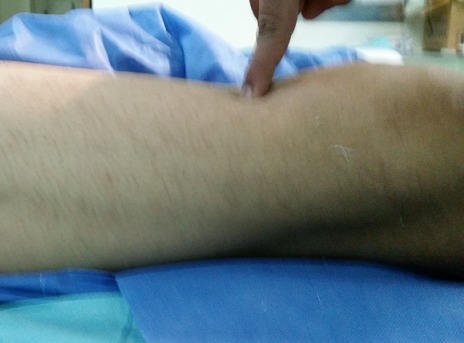
Aspect clinique avec dépression objectivée à la pression

**Figure 2:**
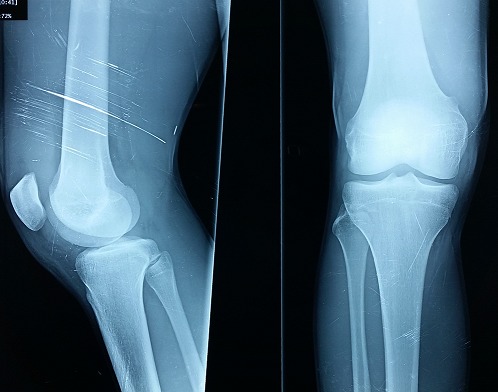
Aspect radiologique

Il s'est agi d'un malade de sexe masculin, âgé de 19 ans, droitier, qui était admis aux urgences traumato-orthopédiques pour impotence fonctionnelle douloureuse brutale du membre inférieur droit des suites d'un accident de football survenant une heure avant son admission. Le patient a été victime d'un choc direct sur la cuisse droite, pied bloqué, avec projection du corps vers l'arrière. Il n'avait pas d'antécédent de maladie chronique systémique, ni de notion de traumatisme antérieur, ni de prise médicamenteuse. L'examen initial a noté un patient en bon état général de poids normal avec un indice de masse corporelle à 22,5 kg/m^2^. L'examen locomoteur local a noté une dépression sus-patellaire douloureuse à la palpation (Figure 1), un syndrome d’épanchement liquidien du genou droit en rapport avec une hémarthrose, un déficit d'extension active du genou droit. Il n'y avait pas d'ouverture cutanée, ni d'atteinte vasculo-nerveuse. Une radiographie standard du genou droit avait objectivé un épaississement des parties molles sus-patellaires sans solution de continuité osseuse (Figure 2).

**Figure 3:**
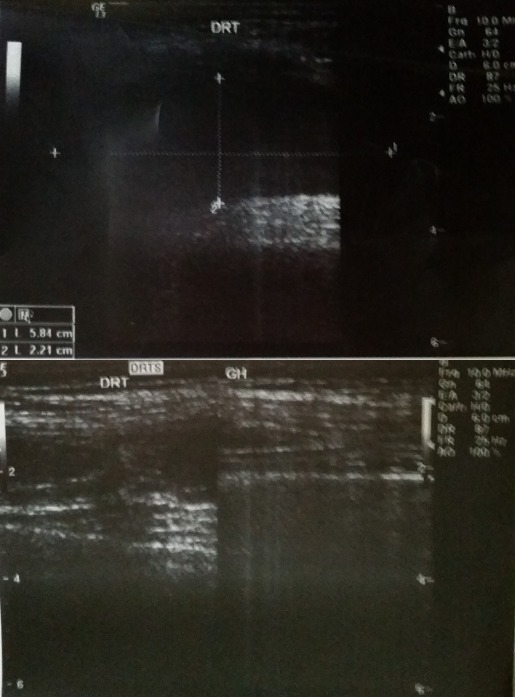
Aspect échographique

**Figure 4:**
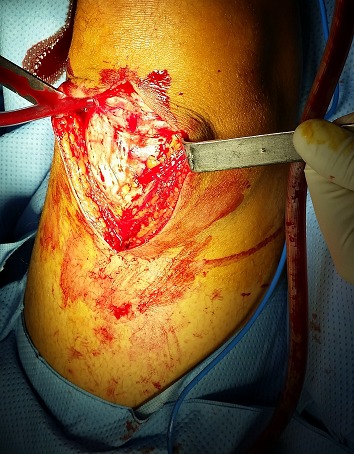
Rupture des muscles droit fémoral et vaste interne après abord chirurgical

**Figure 5:**
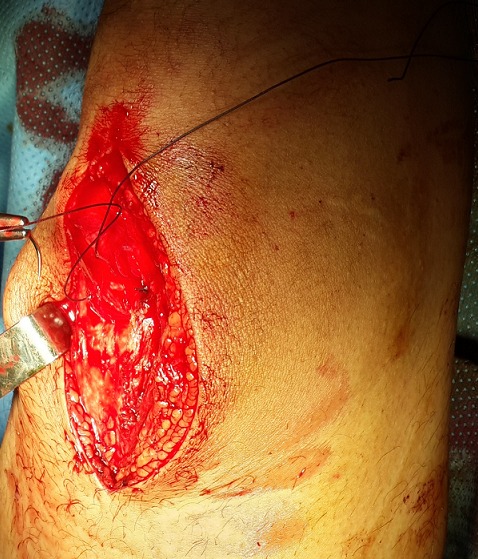
Réparation chirurgicale

L’échographie des parties molles de la cuisse et du genou droits avait noté une discontinuité transversale des fibres du ligament quadricipital à 3 cm de son insertion patellaire, associée à un épanchement articulaire (Figure 3). Après mesures physiques par glaçage, antalgiques par voie intraveineuse, le patient a été opéré en urgence. L'abord chirurgical a été antérieur médian de la cuisse droite, sus-patellaire en regard de la dépression. L'exploration chirurgicale a retrouvé un hématome sous-fascial, une rupture des fibres musculaires du vaste médial et du droit fémoral (Figure 4), une hémarthrose. Nous avons procédé par une suture musculaire par du fil résorbable (Figure 5), une évacuation de l'hémarthrose, lavage articulaire et fermeture sur drain aspiratif articulaire. Une attelle genouillère provisoire a été mise en place pour trois semaines. La rééducation fonctionnelle active du genou avec renforcement musculaire et amélioration des amplitudes articulaires ont débuté ensuite, pendant six semaines. Le genou a regagné ses amplitudes normales. Le score KSCRS (Knee Society Clinical Rating System) [3] évalué à 2 mois était excellent à 98. Ce score permettant d'apprécier douleur, force musculaire et fonction du genou.

## Discussion

La rupture sous-cutanée du ligament quadricipital est moins fréquente, se révèle souvent après 40 ans et résulte presque toujours de facteurs prédisposants tels l'insuffisance rénale chronique, le diabète sucré, la goutte, l'hyperparathyroïdie secondaire ou tertiaire [1, 2]. *Balik et al* ont rapporté un cas de rupture du tendon quadricipital des suites d'une crise épileptique [4]. La prédominance masculine et la rupture sur membre non dominant seraient plus fréquemment rencontrées [5]. Notre patient était jeune sans pathologie chronique prédisposante. Chez les sportifs, la rupture est souvent bilatérale et fait suite à des sollicitations répétées des muscles quadricipitaux [6], ou suite à une prise de stéroïdes anabolisants [7]. N’étant pas sportif de compétition, notre patient serait donc indemne de microtraumatismes répétés. Il est n'a signalé aucune prise médicamenteuse. Le traumatisme a été indirect et violent, expliquant l'unilatéralité de la lésion. L'hyper-extension contrariée avec pied bloqué a été le mécanisme en cause chez notre patient. Le diagnostic doit être établi sur la base des données cliniques notamment la douleur et l'incapacité d'extension active du genou. L'imagerie par résonnance magnétique nucléaire mais aussi l’échographie [8, 9] permettent de confirmer le diagnostic mais ne doivent pas le retarder. La place de la radiographie n'est pas à négliger. Elle recherche un épaississement des parties molles sus-patellaires et aussi une solution de continuité osseuse dans le cadre d'une avulsion de l'insertion patellaire. La réparation reste toujours chirurgicale [1, 2]. Elle doit se faire dans les 72 heures car le résultat fonctionnel peut être compromis si le geste chirurgical est pratiqué 5 à 7 jours après le traumatisme [10]. Il y a donc nécessité de ne pas retarder le diagnostic par quelconque moyen d'imagerie vu que celui-ci reste majoritairement clinique [10]. La rééducation fonctionnelle est obligatoire, adapté au patient et à la douleur. Elle vise le renforcement musculaire et les amplitudes articulaires.

## Conclusion

La rupture sous-cutanée traumatique du sujet jeune est rare mais pas exclue tel que démontre notre dossier. Le diagnostic doit se faire en urgence et ne nécessite pas toujours une imagerie de dernière génération. La prise en charge chirurgicale adéquate en urgence associé à un protocole de rééducation fonctionnel adapté, permet une récupération fonctionnelle complète et une satisfaction du patient.
